# Challenges and Prospects for Preventing the COVID-19 Pandemic in Nursing Homes in Taiwan: A Mixed-Methods Study

**DOI:** 10.1097/jnr.0000000000000757

**Published:** 2026-08-13

**Authors:** Po-Jen KUNG, Kuei-Min CHEN, Ying-Ying CHANG, Li-Yin CHANG, Ching-Min CHEN, Jing-Jy WANG

**Affiliations:** 1 Alice Lee Centre for Nursing Studies, Yong Loo Lin School of Medicine, National University of Singapore, Singapore; 2 Department of Nursing, College of Nursing, Kaohsiung Medical University, Kaohsiung, Taiwan, ROC; 3 Department of Nursing, Taichung Veterans General Hospital Puli Branch, Taiwan, ROC; 4 Department of Nursing, Taichung Veterans General Hospital, Taichung, Taiwan, ROC; 5 Department of Nursing, Central Taiwan University of Science and Technology, Taiwan, ROC; 6 Department of Nursing, College of Medicine, National Cheng Kung University, Tainan, Taiwan, ROC; † Contributed equally

**Keywords:** COVID-19, emerging infectious diseases, nursing homes, pandemic, policy advocacy

## Abstract

**Background:**

During the COVID-19 pandemic, residents of Taiwanese nursing homes, many of whom suffered from chronic illnesses and lived in relatively enclosed environments, were particularly vulnerable to infection. The absence of clear and effective pandemic prevention strategies hindered the development of related risk-reduction policies and planning within these institutions.

**Purpose:**

The aim of this study was to: (1) explore the challenges and prospects related to COVID-19 pandemic prevention in nursing homes in Taiwan and offer recommendations for the future, (2) evaluate the effectiveness of the prevention strategies implemented across nursing homes of various types and scales, and (3) assess the need for pandemic-related institutional training.

**Methods:**

In this exploratory sequential mixed-methods study, a focus group discussion was first conducted to identify key challenges and recommendations, which were then used to inform the development of a quantitative survey. The survey was administered using stratified random sampling based on institution type and scale. Thematic analysis was used for qualitative data, and descriptive statistics, *t*-tests, analysis of variance, and multiple linear regression were applied to analyze the quantitative findings.

**Results:**

Ten pandemic-prevention leaders participated in the focus group discussion, which revealed the four key themes of “Adapting to evolving and unfamiliar roles,” “Being overwhelmed by exhaustion during the sudden pandemic,” “Lack of competence in nonroutine care,” and “Comprehensive and systematic approach at all levels.” The quantitative survey yielded 253 valid responses. The average score for Strategies for Prevention Effectiveness was 28.1 (scale range=9–36), indicating a generally positive self-assessment. However, 6.8% of pandemic prevention supervisors (17 of the 253 participants) had not received COVID-19-related training. The analysis highlighted the importance of incorporating successful prevention experiences from other institutions and providing frontline health care workers with education on pandemic-related regulations. The average score for Educational Training was 16.2 (scale range=5–20), reflecting strong agreement with the listed training items. No significant differences were found in pandemic strategy effectiveness by either institution type (*t*=1.664, *p*=.097) or scale (*F*=0.571, *p*=.566).

**Conclusions/Implications for Practice:**

The results show generally positive self-assessments of prevention strategies but revealed key challenges, including role confusion, frontline exhaustion, and training gaps. Although the World Health Organization has declared COVID-19 to no longer be a public health emergency of international concern, these issues remain critical to institutional preparedness. The findings offer transferable insights that may inform future pandemic response planning in long-term care settings, regardless of the nature of the infectious threat.

## Introduction

Taiwan became a super-aged society in 2025, with adults aged 65 years and older accounting for 20.06% of the total population by the end of the year (Ministry of the Interior, 2026). This demographic shift, combined with declining birth rates and weakened intergenerational caregiving networks, has led to a growing demand for institutional long-term care services (Liu & Chi, 2019). Among long-term care institutions, nursing homes play a critical role in providing care to older individuals with severe functional impairments who often require assistance with daily activities and rely on advanced medical equipment and specialized care.

Nursing home residents were disproportionately affected by COVID-19 due to their advanced age, chronic conditions, and the close-contact nature of institutional living (Gao et al., 2021; Shrader et al., 2021; Yi et al., 2020). The physical layout and operational routines in nursing homes, for example, shared bathrooms and dining spaces, and frequent interaction between staff and residents, present multiple transmission pathways (ASPR TRACIE, 2023; Henriques et al., 2023). Also, many frontline workers during the COVID-19 pandemic lacked adequate training in infection prevention, leading to moral distress and uncertainty about whether their care met professional expectations (Birt et al., 2023). The limited physical space in many facilities further hindered zoning and cohorting strategies, complicating containment efforts (Benjamin et al., 2014; Corpora et al., 2021).

A recent systematic review highlighted that the effectiveness of infection prevention measures in long-term care varies across settings (Bloch et al., 2023). While that review did not analyze institutional scale explicitly, several of the included studies describe differences in outcomes among facilities of different scales, types (e.g., nursing homes vs. assisted living), staffing levels, and infrastructures, suggesting these organizational factors may influence infection prevention and control implementation and success. However, many nursing homes remain under-resourced and equipped with only basic medical tools, increasing the burden on care teams during health emergencies (Anesi & Kerlin, 2021). Despite these known vulnerabilities, few empirical studies in Taiwan have been conducted to examine how the response of nursing homes to the COVID-19 pandemic differed based on type and scale or how frontline workers perceived and evaluated the enacted prevention strategies. Evidence is also limited on whether staff felt adequately trained to manage the emerging infectious threats. These gaps highlight a need for context-specific knowledge to inform policy and strengthen preparedness in long-term care.

This study was designed to examine the infection-prevention-related challenges and preparedness gaps faced by nursing homes in Taiwan during the COVID-19 pandemic. The focus was on the effectiveness of the implemented prevention strategies and differences in these strategies across institutional types (i.e., independent vs hospital-affiliated) and scales, as well as the educational and training needs of staff. By identifying key lessons and future directions, the aim was to inform more resilient and equitable infection prevention planning in long-term care.

## Methods

### Research Design

An exploratory sequential mixed-methods design was used to first explore frontline experiences and then validate findings in a larger sample. Next, a semi-structured focus group discussion with senior health care workers was used to identify the challenges and suggestions related to COVID-19 prevention in nursing homes. The qualitative findings were transformed into questionnaire items systematically using an item generation process that involved: (1) mapping emergent themes to potential survey domains, (2) drafting an initial pool of items based on representative participant quotes and relevant literature, and (3) conducting an expert review and pilot testing before finalization. These findings were used in creating a cross-sectional questionnaire that was applied to assess strategy effectiveness and training needs across institutions of differing types and scales.

### Participants and Sample

The study population comprised health care workers working at nursing homes registered with the Ministry of Health and Welfare of Taiwan. The nursing homes were categorized into two types (independent and hospital-affiliated) and three scales (small: <50 beds; medium: 51–100 beds; and large: >100 beds).

In the qualitative phase, purposive sampling was used to recruit senior health care workers with at least 5 years of experience who were fluent in Mandarin and willing to provide informed consent. A total of 10 participants (1–2 from each combination of institutional type and scale) were selected, ensuring an adequate diversity of perspectives.

In the quantitative phase, stratified random sampling was applied using the national nursing home registry, which includes 360 independent and 136 hospital-affiliated small (129), medium (275), and large (92) institutions. The institutions were selected randomly within each stratum, with one pandemic prevention supervisor from each invited to participate in the survey. The eligibility criteria included: active employment during the survey period, involvement in COVID-19 prevention efforts, and proficiency in reading traditional Chinese.

The minimum sample size was calculated using two methods. Firstly, for the inferential analyses, an initial estimation for multiple regression was conducted using G*Power (Faul et al., 2009). Assuming a medium effect size (*f* ^2^=0.15), significance level of α=.05, statistical power of 0.80, and a maximum of two predictors, the minimum sample size was determined as 55 to ensure adequate power to detect meaningful associations in the regression model. Secondly, a sample size calculation based on a confidence level of 95%, a confidence interval of ± 5%, and a population of 518 nursing home supervisors in Taiwan (Creative Research Systems, n.d.) was used, yielding a minimum sample size of 221. To account for potential nonresponse (~20%), the recruitment target was set at 265. The final valid sample comprised 39 small, 93 medium, and 26 large independent nursing homes, and 20 small, 27 medium, and 16 large hospital-affiliated nursing homes.

### Instruments

In the qualitative phase, the semi-structured interview guidelines were refined using the results of expert consultations conducted based on the study objectives and a literature review. Interview questions included: (1) What were your institution’s experiences during the COVID-19 pandemic? (2) What challenges did your institution face during the peak of the pandemic? (3) What suggestions do you have for future emerging infectious disease prevention? Follow-up questions, for example, asking for specific examples, clarification, and elaboration on institutional strategies and staff experiences, were used to explore the responses in greater depth.

In the quantitative phase, two independent scales were developed. The initial item pool (14 items: 9 in the Strategies for Prevention Scale and 5 in the Educational Training Scale) was drafted using the identified qualitative themes and then cross-checked against existing guidelines and prior related research (Kung et al., 2023). Next, ten experts, two in infectious diseases, two in long-term care administration, and two in nursing education, assessed each item for relevance, clarity, and comprehensiveness. Content validity was quantified using the content validity index (CVI), yielding CVI values of .97 and .94 for the two scales, respectively. The pilot test included 15 pandemic prevention supervisors from diverse institutional types and scales to ensure clarity and feasibility. This sequential design helped ensure the qualitative findings informed the quantitative instrument, while expert review ensured content validity.

### Data Collection

Data collection for the questionnaire survey was conducted from November 2023 to January 2024. A focus group discussion was audio-recorded with the consent of the participants and transcribed within two days. A senior nursing scholar moderated the discussion, with researchers trained in qualitative research assisting as observers and note-takers. Before data summarization, the discussion was concluded when no new themes emerged, and the moderator confirmed that all participants had fully expressed their ideas. The research team reviewed findings immediately after the discussion. To ensure trustworthiness, methodological rigor was maintained following the criteria of Lincoln and Guba (1986), credibility was supported by clearly communicating research objectives and ensuring full participation, dependability was enhanced using a team-based review of the findings, and confirmability was ensured using timely transcription and the systematic documentation of procedures. For the quantitative survey, anonymous paper questionnaires were distributed after obtaining institutional consent. Data collection continued until the target sample size was reached.

### Data Analysis

Thematic analysis was used to analyze the qualitative data, as follows: (a) data familiarization, (b) theme identification, and (c) theme organization (Sundler et al., 2019). The research team facilitated the analysis using NVivo version 14.23.0 (Lumivero, Denver, CO, USA), with coding performed independently by two authors and reviewed by a qualitative research expert. Participant feedback was solicited by sharing the summary of preliminary themes to validate the results, with minor wording adjustments made based on this feedback.

The quantitative data were analyzed using IBM SPSS Statistics version 27.0 (IBM Corp., Armonk, NY, USA), and statistical tests were conducted using *p*<.05 as the threshold for significance. Continuous variables were characterized using means and standard deviations. *T*-tests and analyses of variance were used to compare the effectiveness of prevention strategies employed by institutions of different types and scales. Finally, multiple linear regression was employed to identify significant predictors of prevention strategy effectiveness across different institutional types and scales.

### Ethical Approval

Data collection commenced after approval was received in May 2023 from the institutional review board of National Cheng Kung University Hospital (Approval No. B-ER-112-090), with the first participant recruited in July 2023. The participants were fully informed of the study purpose, ethical principles, and questionnaire contents. Data collection was conducted only with informed consent. Ethical approval and contact information for the ethics review committee were provided for participant consultation.

## Results

The focus group conducted with frontline nursing home leaders yielded the four core themes of role adaptation, emotional burden, nonroutine care demands, and the need for systemic preparedness. These themes were systematically translated into survey constructs to assess their broader relevance across institutions. Thus, in the quantitative phase, the validity and generalizability of these qualitative insights were demonstrated, with these results reinforcing the urgency for structural reforms in pandemic preparedness. The detailed findings from both the qualitative and quantitative phases are presented below.

### Qualitative Results

A focus group discussion was conducted in August 2023 to explore the challenges and future prevention recommendations related to the COVID-19 pandemic in nursing homes. Ten participants (all female leaders with nursing backgrounds and an average tenure of 6.9±6.8 years) participated together in a single discussion session. All were currently responsible for pandemic prevention at their respective institutions and well-versed in planning and managing COVID-19 measures. Four key themes and corresponding sub-themes emerged from the discussion, as summarized in Table 1.

**Table 1:** COVID-19 Prevention Challenges in and Recommendations for Nursing Homes, Themes, and Sub-Themes

Theme	Sub-Theme
Adapting to evolving and unfamiliar roles	1. Emotional support providers
	2. Consultants for residents and family members
	3. Team coordinators
	4. Educators for residents, families, and staff
Being overwhelmed by exhaustion during the sudden pandemic	1. Empathy and care
	2. Communication and cooperation
	3. Physical and emotional toll
Lack of competence in nonroutine care	1. Managing informatics and telehealth literacy
	2. Innovative thinking
	3. Data processing and analysis
	4. Cross-disciplinary knowledge transfer
Comprehensive and systematic approach at all levels	1. Tangible resources
	2. Intangible resources
	3. Routine practices
	4. Policy compliance
	5. Successful experience sharing

The first three themes highlight the main challenges encountered during the pandemic. These are (1) adapting to evolving and unfamiliar roles, (2) being overwhelmed by exhaustion during the sudden pandemic, and (3) lack of competence in nonroutine care. The fourth theme, which relates to future prevention recommendations, emphasizes the need for a comprehensive and systematic approach at all levels to better prepare nursing homes for future infectious disease crises. These themes are described individually as follows:

### Theme 1: Adapting to Evolving and Unfamiliar Roles

Health care workers in nursing homes serve as frontline responders and are key pillars in pandemic prevention. Their roles continuously evolved through the pandemic period, with new and often unfamiliar responsibilities placing additional demands on their emotional, organizational, and educational capacities. For example, during strict lockdown periods, they often took on roles similar to those of family members to provide emotional support to residents. In response to unexpected cases or outbreaks, they became coordinators, managing complex internal and external communications. Furthermore, they took on the role of consultants and educators, providing guidance to both residents and their families, while also training administrative staff on critical pandemic prevention measures.

#### 1. Emotional support providers

Due to visitor restrictions, residents faced long periods without family contact, leading to feelings of isolation and distress. Staff stepped in to fill this emotional gap, providing psychological support that extended beyond routine care and helping residents cope.

*“When their families couldn’t visit, we became like family to the residents. We listened, comforted them, and supported them emotionally in any way we could.”* (#03 Assistant Head Nurse)

#### 2. Consultants for residents and family members

During the pandemic, uncertainty left residents and their families anxious. Staff provided essential information and reassurance, maintaining trust and easing concerns, particularly during severe outbreaks.

*“Isolated patients felt helpless, but we could still talk to them through microphones. Even while wearing full protective gear, we could hold their hands during treatment and provide consultation and answers to their questions. During severe outbreaks, we also received countless calls every day from anxious family members needing reassurance and explanations.”* (#07 Head Nurse)

#### 3. Team coordinators

Health care workers coordinated operational tasks to manage clinical care while addressing the emotional and logistical challenges brought by confirmed cases.

*“A confirmed case was not just a number; it involved a lot of operations, including requesting support for moving residents, promptly reporting to supervisors, contacting families, and notifying doctors.”* (#09 Director of Nursing)

#### 4. Educators for residents, families, and staff

Staff ensured residents, administrative staff, and families were informed about preventive measures, providing necessary guidance on how to self-protect and protect others.

*“In addition to educating residents, we needed to train administrative staff on the correct prevention measures to protect themselves and explain visitor restrictions to the families.”* (#02 Institution Administrator)

### Theme 2: Being Overwhelmed by Exhaustion During the Sudden Pandemic

Health care workers faced overwhelming physical and mental exhaustion as they navigated the unpredictable challenges posed by the COVID-19 pandemic. The constant demands of providing care, managing communication with anxious families, and coordinating emergency responses left many workers feeling drained. Although they attempted to show empathy and care for the residents and their families, the sheer intensity of the pandemic took a toll on their emotional well-being, especially when feelings of helplessness, anxiety, and fear set in.

#### 1. Empathy and Care

When family-resident communications were cut off or severely restricted, the residents were upset, and their family members expressed frustration and anger, often through persistent complaints. Staff found that, while listening helped ease family anxieties, managing ongoing grievances contributed to their own emotional fatigue.

*“During visitor restrictions, residents were sad not to see their families, and families were angry and often called the nursing station to complain. Listening to their outbursts helped, but dealing with these long-term complaints took a toll on my mood and left me feeling overwhelmed.”* (#06 Head Nurse)

#### 2. Communication and cooperation

Staff were required to coordinate with health authorities, recall off-duty colleagues to work, secure protective equipment, and implement visitor restrictions, all while dealing with the immense pressures of the situation.

*“When an outbreak occurred in the unit, we needed everyone’s effort. We had to call people on leave to come back, confirm contacts and tests with the health bureau, secure enough protective equipment, and announce visitor restrictions. Honestly, I felt both physically and mentally drained.”* (#10 Nursing Supervisor)

#### 3. Physical and emotional toll

The sudden appearance of COVID-19 cases necessitated an immediate emergency response. Staff worked under immense pressure, fearing for their own safety while still performing their duties. The ongoing stress and lack of recovery time left them physically and emotionally exhausted.

*“When our first resident tested positive, it was chaotic, and we had to activate the emergency plan immediately. I was scared, but I had to do my job. Every day was busy and exhausting, and there was no time to fully recover from the stress.”* (#03 Assistant Head Nurse)

### Theme 3: Lack of Competence in Nonroutine Care

Health care workers were well-trained in routine care activities such as assessing symptoms, recognizing infectious diseases, and selecting proper protective equipment. However, the sudden demand for nonroutine skills during the pandemic exposed a significant gap in their preparedness. Many found themselves overwhelmed by the rapid shift to informatics and telehealth, which were unfamiliar technologies that became crucial in care provision during the pandemic. This lack of familiarity often led to mistakes and delays in communication, increasing the burden on both staff and residents. Furthermore, innovative thinking was required to implement new care strategies, yet many staff felt unprepared for this level of problem-solving. Managing confirmed cases also required knowledge beyond traditional care skills, further compounding the stress and confusion already present among staff.

#### 1. Managing informatics and telehealth literacy

The rapid adoption of virtual consultations posed significant challenges. Staff struggled to keep up with the learning curve, often unsure how to proceed when the conditions of residents suddenly changed. This unfamiliarity caused delays in care and increased the complexity involved in managing residents’ health.

*“We were not used to using virtual media for consultations, and it took time to adjust. The learning curve was steep, and sometimes, we were unsure how to proceed when residents’ symptoms changed suddenly.”* (#01 Director of Nursing)

#### 2. Innovative thinking

The pandemic required creative approaches to care, but many staff felt unprepared to think outside the box.

*“Innovative care methods such as adjusting food presentation to increase appetite were necessary, but not everyone had the skills to think outside the box. We also struggled to adopt large-scale disinfection procedures like homemade hypochlorous acid.”* (#04 Nursing Supervisor)

#### 3. Data processing and analysis

The volume of data generated by tracking infection sources and managing pandemic responses was overwhelming. Staff lacked the expertise to handle complex data, leading to delays in decision-making and increased pressure.

*“Tracking and analysing data to identify infection sources was challenging. We lacked the expertise to handle the volume of information, which sometimes delayed important decisions.”* (#10 Nursing Supervisor)

#### 4. Cross-disciplinary knowledge transfer

In addition to care skills, keeping up with constantly changing pandemic-related regulations, laws, and vaccination updates proved difficult.

*“Aside from care skills, we found it difficult to keep up with constantly changing pandemic-related laws and vaccination updates. Families and residents had many questions, but we weren’t always sure how to answer them.”* (#06 Head Nurse)

### Theme 4: Comprehensive and Systematic Approach at All Levels

Promoting emergent infectious disease prevention in institutions requires a systematic and comprehensive approach. Five key elements were identified during the discussion: tangible resources, intangible resources, routine practices, policy compliance, and successful experience sharing.

#### 1. Tangible resources

Adequate supplies were critical, but shortages strained staff efforts to balance care quality and well-being.

*“Backup support is crucial during a pandemic. We faced shortages of masks and protective clothing because many suppliers were out of stock, and scheduling was challenging when you have to balance care quality and staff well-being.”* (#08 Nursing Supervisor)

#### 2. Intangible resources

Cooperative relationships with long-term residents and supportive leadership helped foster a more collaborative environment, despite the challenges posed by newer, less cooperative residents.

*“Long-term residents, who were like family, understood our hard work and cooperated more. Newer residents were often more resistant, making it harder to implement preventive measures. Although it was not easy, having a solid connection with the institution’s philosophy and the supervisors’ openness to our suggestions created a rather collaborative environment, which made me more willing to work with them.”* (#07 Head Nurse)

#### 3. Routine practices

Regular hand hygiene audits and vaccination efforts, driven by lessons learned from outbreaks in other institutions, helped maintain infection control.

*“Regular hand hygiene audits were essential. Hearing about outbreaks in other institutions alarmed me, encouraging me to review our prevention plans and conduct training sessions. We also encouraged our staff to get vaccinated. On a personal level, I avoided crowded places during the pandemic. If I had symptoms, I would wear a mask and check if I had been in contact with any confirmed cases.”* (#05 Institution Administrator)

#### 4. Policy compliance

Frequent updates to national guidelines required staff to stay informed and adapt quickly to new policies on visitor restrictions, testing, and isolation.

*“Policies from the command centre changed frequently, requiring constant updates to visitor rules, staff testing frequencies, and resident isolation locations. The continuously evolving pandemic and virus mutations meant we had to continuously update staff on the latest CDC care guidelines and diagnostic standards.”* (#09 Director of Nursing)

#### 5. Successful experience sharing

Sharing best practices between institutions allowed staff to apply effective, practical prevention strategies in real time.

*“Training had to be practical and immediately applicable. Sharing successful prevention experiences among institutions provided valuable insights and training for staff.”* (#08 Nursing Supervisor)

### Quantitative Results

Two hundred sixty-five nursing homes were randomly selected, and 256 questionnaires were returned (response rate of 96.6%), with one pandemic prevention supervisor from each institution invited to complete the questionnaire. After excluding 3 invalid questionnaires (incomplete responses) based on a stratified sampling plan, 253 valid questionnaires were deemed valid and were used in the analysis. The participants worked at either independent nursing homes (43 small, 97 medium, and 28 large) or hospital-affiliated nursing homes (27 small, 35 medium, and 23 large). The largest group of respondents was institution managers (41.9%), followed by head nurses (18.2%), nursing staff (9.5%), social workers (4.3%), and others (26.1%). The average bed occupancy rate reported by the participating institutions during the COVID-19 pandemic (2022–2023) was ∼87.5%. These distributions captured the intended representativeness across institutional types and exceeded the minimum stratum-specific sample sizes defined in the proportional stratified sampling plan.

The Strategies for Prevention taken by personnel showed an average score of 28.1 (scale range=9–36), indicating a generally positive outcome, although with room for improvement in some areas. The highest-scoring item was “The institution has established channels for announcing the latest pandemic information to residents or families, such as LINE groups and video calls” (average score: 3.63). The lowest-scoring item was “It is easy to transfer infected residents to acute medical care” (average score of 2.55). This suggests that, although the nursing homes were proactive in creating communication channels to disseminate pandemic prevention information, significant challenges existed in the systems used to transfer infected residents for medical care. Notably, no item had an average score below 2, reflecting a generally positive outcome of the pandemic prevention strategies implemented by the supervisors. The detailed score distributions for each item are provided in Table 2.

**Table 2:** Prevention Strategies (*N*=253)

Item Number	Item Description	Mean (*SD*)	Score Order
1	Staff are knowledgeable about the institution’s inventory of prevention supplies.	3.19 (0.54)	4
2	Staff can effectively implement the institution’s visitation guidelines.	3.55 (0.51)	2
3	Staff can promptly provide residents or family members with updates on the COVID-19 situation, including prevention policies and compliance measures.	3.46 (0.54)	3
4	The institution adjusts care practices based on operational considerations.	2.94 (0.72)	7
5	The institution has established channels for announcing the latest pandemic information to residents or families, such as LINE groups and video calls.	3.63 (0.55)	1
6	The institution can efficiently adjust staffing and scheduling.	3.14 (0.68)	5
7	It is easy to transfer infected residents to acute medical care.	2.55 (0.98)	9
8	The institution has stockpiled sufficient supplies for emergency preparedness.	3.07 (0.72)	6
9	The institution has channels to propose prevention-related policy recommendations to government authorities.	2.64 (0.80)	8

The Educational Training needs assessment revealed that most of the participants had received on-the-job training on COVID-19, although 6.8% had not participated in any related courses. The analysis showed an average total score of 16.2 (scale range=5–20), indicating strong agreement with the listed educational training items among the pandemic prevention supervisors. The items “Learning from the successful COVID-19 prevention experiences of other institutions is important” and “It is necessary for health care professionals to acquire knowledge about pandemic-related laws” both received the highest average score of 3.62. The results of the detailed item score analysis on both scales are provided in Table 3.

**Table 3:** Educational Training (*N*=253)

Item Number	Item Description	Mean *(SD*)	Score Order
1	The COVID-19 prevention training provided by the institution meets the practical needs of prevention efforts.	3.21 (0.66)	4
2	Health care professionals should have access to educational information related to COVID-19 vaccines.	2.74 (0.49)	5
3	The institution or government should adjust health care professionals’ annual training hours in response to pandemic developments.	2.97 (0.34)	3
4	Learning from the successful COVID-19 prevention experiences of other institutions is important.	3.62 (0.66)	2
5	It is necessary for health care professionals to acquire knowledge about pandemic-related laws.	3.62 (0.75)	1

Notably, no significant differences were found in the effectiveness of prevention strategies either between hospital-affiliated and independent nursing homes (*t*-test value of 1.664, *p*=.097) or among nursing homes of different scales (*F*-test value of 0.571, *p*=.566).

## Discussion

Although the findings span multiple dimensions, they address the three guiding aims of this study. The results of the qualitative analysis revealed four core challenges in institutional pandemic response: shifting staff roles, emotional strain, limited preparedness for nonroutine care, and the need for system-level coordination. The quantitative results showed no significant differences in prevention effectiveness across nursing home types or sizes, suggesting broad consistency. Although most staff had received pandemic-related training, a small but notable proportion had not, pointing to persistent gaps in workforce readiness. Conducted in May 2023, this mixed-methods study retrospectively examined COVID-19 prevention efforts in Taiwanese nursing homes and, despite the WHO declaration that COVID-19 is no longer a Public Health Emergency of International Concern, the ongoing threat of emerging infectious diseases underscores the relevance of these findings for future preparedness in long-term care settings.

The study revealed that the health care workers’ roles and competencies in nursing homes during the pandemic were dynamic, as they were expected to adapt continuously to the unpredictable nature of an emerging infectious disease. This finding is consistent with a review study focused on health care workers in long-term care institutions engaged in emerging infectious disease prevention (Kung et al., 2023). However, the evolving responsibilities and competencies during the pandemic led to perceptions of unfairness and dissatisfaction among health care workers (Kackin et al., 2021), which indirectly increased the complexity of resident care (Montayre & Montayre, 2017). The results show that the infection prevention goals evolved with the evolving pandemic situation. Initially, zero infections were the goal. But, as the pandemic progressed, institutions adjusted the goal to be living with the virus and preventing cluster infections, necessitating adjustments to current strategies. The emergence of new infectious diseases and pathogen mutations continually influenced infection prevention strategies and nursing home measures, leading inevitably to shifts in the roles of health care workers. This situation also resonates with the findings for the themes “lack of competence in nonroutine care” and “being overwhelmed by exhaustion during the sudden pandemic,” under which participants expressed feelings of being unprepared and overwhelmed by the nonroutine tasks required during the pandemic, highlighting the significant struggles and stresses faced by health care workers as they navigated new responsibilities under high pressure.

Regarding the challenges of and strategies for managing pandemic prevention in nursing homes, most of the participants expressed confidence in the pandemic prevention performance of their institutions, which mirrors the findings of a regional study on the competencies of nursing healthcare workers in long-term care institutions in terms of preventing emerging infectious diseases (Kung & Chen, 2022). However, the relatively high frequency of COVID-19 outbreaks experienced in Taiwanese nursing homes (The Reporter, 2022) highlights a potential discrepancy between the survey results in this study and clinical realities. Possible reasons include overconfidence in pandemic prevention abilities and the retrospective nature of the survey. Moreover, the gap in time between the timing of the qualitative focus group discussion and the quantitative survey may have introduced bias due to evolving circumstances over time. These factors should be considered when interpreting the findings. Moreover, pressure from high public expectations driven by media coverage (Cheng, 2023; Haldane et al., 2021) may have increased self-reporting bias. The more effective utilization of social media to report accurate and positive prevention information may help reduce stigma and strengthen family support for health care workers.

In addition, the qualitative focus group discussion conducted in this study revealed that many families were unaware of their institutions’ COVID-19 prevention measures, contrary to the quantitative survey findings indicating that institutional communication channels were effective. This discrepancy suggests that improving communication skills may enhance family understanding of clinical care information (Razai, 2018). Strengthening the communication and coordination capabilities of health care workers is recommended to ensure the effective transmission of pandemic prevention information. Establishing a centralized prevention information channel may also ensure a clear understanding of and adherence to guidelines.

Together, the qualitative and quantitative findings converge to reveal a systemic picture of the evolving challenges in nursing home pandemic preparedness. The emotional burden and shifting roles described in the focus group discussion were not isolated cases but, rather, problems reflected throughout the survey, particularly with regard to items related to communication and emotional support responsibilities. These results are consistent with international evidence showing that long-term care staff were often expected to manage both caregiving and crisis response roles during the pandemic, often without adequate support (Stall et al., 2020). Similarly, the lack of preparedness in nonroutine care capabilities, for example, telehealth use and legal literacy, was reinforced by being the highest-rated training need on the survey, echoing calls for expanded competencies in long-term care settings (McGilton et al., 2021). The consistency observed across both phases of this study highlights the magnitude and generalizability of these issues, emphasizing the need for structural reform in training, staffing, and institutional preparedness.

Furthermore, no significant differences in COVID-19 prevention strategies were identified by institutional type or scale, which is contrary to previous research that highlighted institutional type as a significant factor of influence on prevention competencies (Martin, 2011). Our focus on nursing homes with relatively consistent resident conditions may help explain the lack of observed differences, suggesting the results for other long-term care institution types may differ due to varying resident conditions.

Previous research has shown that on-the-job education enhances health care workers' knowledge in specific areas (Rentala et al., 2021; Salim et al., 2019). Despite the severe impact of COVID-19 on nursing homes, 6.8% of the participants reported not receiving related training, indicating a need for mandatory education. Revising regulations to increase required infection control training hours for health care staff and enhancing emerging infectious disease knowledge in health care program curricula for students are suggested. Moreover, related programs should incorporate successful prevention practices from high-performing institutions and cover essential knowledge of pandemic-related laws and regulations to enable staff to respond more confidently in crisis scenarios. Furthermore, ensuring institutional leadership is involved in training may boost morale and prevention effort effectiveness.

Finally, the findings emphasize the importance of integrating prevention resources and strategies systematically. Although individual efforts are crucial, the successful prevention of COVID-19 in institutions, as highlighted in other studies, relies on adequate manpower, protective equipment, education, and training, as well as the implementation of effective policies (Cavalcante de Oliveira et al., 2023; Moreland et al., 2023). Institutional leadership involvement impacts pandemic management significantly, as shown in Eckardt et al. (2020), emphasizing the need for mobilizing health care workers at all levels.

### Limitations

In the qualitative phase of this study, senior health care workers were invited via purposive sampling. However, the sample lacked participants from institutions located in Taiwan’s East Coast region due to logistical and scheduling challenges. In future studies, hybrid in-person and online meetings may be used to gain a more comprehensive comparison of experiences across different regions.

Also, the anonymous paper-based surveys used in this study required the participants to recall pandemic conditions, increasing the risks of memory bias and inaccuracies. Also, the high degree of social media attention on nurses and COVID-19 health care during the pandemic may have influenced participant responses, potentially leading to an overestimation bias in their responses. In addition, as the data were collected in Taiwan only, the generalizability of the findings to other regions may be limited. Finally, a newly developed, self-designed questionnaire was used in this study. As factor analysis has not yet been conducted, further research is recommended to examine the construct validity of this questionnaire to enhance its applicability and robustness.

### Conclusions

The effect of the COVID-19 outbreak on nursing homes in Taiwan has led to significant and lasting changes in the roles and responsibilities of health professionals. During the pandemic, health care workers faced evolving roles, exhaustion, and expectations to acquire skills in nonroutine care and systematic management approaches. The findings indicate these institutions maintain strong communication channels, although significant issues remain with regard to transferring infected residents for appropriate care. The findings reinforce the importance of all prevention supervisors receiving comprehensive pandemic-related education and training. No significant differences in prevention effectiveness were found between hospital-affiliated and independent nursing homes or among institutions of different scales. Addressing these gaps and leveraging existing strengths are essential to improving future pandemic preparedness, with the results providing clear guidance for policymakers and health care administrators.
